# Genetic diversity using biochemical, physiological, karyological and molecular markers of *Sesamum indicum* L

**DOI:** 10.3389/fgene.2022.1035977

**Published:** 2022-10-12

**Authors:** Salha Mesfer ALshamrani, Fatmah Ahmed Safhi, Dalal Sulaiman Alshaya, Amira A. Ibrahim, Hassan Mansour, Diaa Abd El Moneim

**Affiliations:** ^1^ Department of Biology, College of Science, University of Jeddah, Jeddah, Saudi Arabia; ^2^ Department of Biology, College of Science, Princess Nourah bint Abdulrahman University, Riyadh, Saudi Arabia; ^3^ Botany and Microbiology Department, Faculty of Science, Arish University, El-Arish, Egypt; ^4^ Department of Biological Sciences, College of Science & Arts, King Abdulaziz University, Rabigh, Saudi Arabia; ^5^ Botany Department, Faculty of Science, Suez Canal University Ismailia, Ismailia, Egypt; ^6^ Department of Plant Production, (Genetic Branch), Faculty of Environmental Agricultural Sciences, Arish University, El-Arish, Egypt

**Keywords:** genetic diversity, ISSR, karyotype analysis, SCoT, semsame, *Sesamum indicum* L.

## Abstract

The genetic diversity and the relationships among sesame cultivars were investigated using physiological and cyto/molecular analysis. To our information, no studies have yet been conducted on the genetic evaluation of sesame genotypes based on cyto/molecular analysis in Saudi Arabia. This study showed that genotype Bah-312 had the highest values from physiological and biochemical traits (plant height, harvest index, total plant dry matter, seed yield, oil content, and fatty acids content). Using 20 ISSR and 25 SCoT primers, the studied genotypes amplified 233 and 275 alleles, while the average polymorphism percentage (P%) was 65.32% (ISSR) and 77.8% (SCoT) across all the studied genotypes, respectively. To assess the markers efficiency analysis the polymorphism information contents (PIC), Marker Index (MI), Effective Multiplex Ratio (EMR), Resolving Power (Rp) were estimated. In general, primers (ISSR 2 & SCoT 21) and (ISSR 4 & SCoT 3) revealed the highest and lowest values for P %, PIC, MI, and EMR%. Furthermore, 188 positive and negative unique bands were detected, out of which ISSR generated 84, while 104 were amplified by SCoT analysis. In this regard, genotype Bah-312 generated 41 unique amplicons, and Jiz-511 genotype 23 unique amplicons. In the same context, the population genetics parameters, number of different alleles (Na), number of effective alleles (Ne), Shannon’s index (I), expected heterozygosity (He), and Unbiased Expected Heterozygosity (uHe), were calculated. ISSR marker showed the highest values for all the estimated parameters. In this regard, genotype Bah-312 exhibited the highest values (1.35, 1.37, 0.31, 0.21, 0.29) & (1.31, 1.35, 0.30, 0.20, 0.27) while, genotype Ahs-670 revealed the least values (1.29, 1.31, 0.26, 0.16, 0.23) &(1.14, 1.26, 0.22, 0.15, 0.20) for ISSR and SCoT markers respectively. For cytological data, according to the highest asymmetry index (AsK%) and lowest total form percentage (TF%) values, genotype Ahs-670 was the most advanced cultivar, and genotype Bah-312 was the most primitive one. According to the degree of asymmetry of karyotype (A) and intrachromosomal asymmetry index (A1), sesame genotype Ahs-670 was the most asymmetrical, and Bah-312 was the most symmetrical genotype. This study gives some helpful information about the genetic diversity of six sesame landraces. The variation harbored by these landraces could be used in sesame breeding programs.

## Introduction

One of the most ancient annual oilseed crops cultivated in tropical and temperate regions is Sesame (*Sesamum indicum* L.) (Amoo et al., 2017). It is a self-pollinated diploid plant (2n = 26) with oil-rich seeds (50–60%) and antioxidants (Sharma et al., 2014). The size of the sesame genome, which is around 350 megabytes, has not been extensively studied. In a sesame reference genome with a low amount of repetitive sequences (28.5%), a total of 27,148 genes have been annotated (Wang et al., 2014). Sesame seeds are a protein source, a high-quality edible oil with a high amount of polyunsaturated fatty acids and various other nutrients, including vitamins, minerals, and important antioxidant lignans (sesamin, sesamolin, sesamol) (Dar and Arumugam, 2013; Pathak et al., 2017). Sesame seed oil has long been used for human consumption and various industrial applications.

Recently, it received much attention due to its high oil quality, which contains many oleic and linoleic acids (Dar et al., 2019a). Asia produces 70% of the world’s Sesame, while Africa accounts for the remaining 26%. India, Myanmar, China, Sudan, Uganda, Ethiopia, and Nigeria are the world’s major producers of Sesame (Food and Agriculture Organization of the United Nations, 2018). In Saudi Arabia (KSA), Sesame is an economic crop cultivated in Makkah and Gizan regions (Sher and Hussain, 2009; Alyemeni et al., 2011). Sesame production in KSA is 4.021 tons from 3.056 ha, and the average seed yield is 623 kg ha^−1^ (Ministry of environment water and agricultural, 2021). Saudi Arabis’s Sesame stays undeveloped because of many restrictions, such as uncharacterized plant material and the lack of improved cultivars. This situation can be improved by generating and releasing high-yielding, high-quality varieties with high adaptability to the growing circumstances of this crop (Nyongesa et al., 2013). Previous to that, genetic information on local landraces is required to examine the current genetic diversity.

Estimating genetic diversity is considered a precursor for crop improvement, giving relevant data for selections and breeding programs (Pal et al., 2016; Erdinc et al., 2021; Hasanbegovic et al., 2021). Furthermore, it is helping researchers develop new cultivars with desired traits such as yield and quality; genetic resources facilitate the introgression of novel traits required in the production of plants under different climates (Holmes et al., 2019; Park et al., 2021). Genetic polymorphism in Sesame was established using germplasm pool, phenotyping, and genotyping selections. New traits and gene discovery programs in Sesame are based on maintaining its genetic diversity and the evolution of new sources and landraces with high yield components and resistance to pests and diseases. Moreover, resistance to different abiotic stresses and high nutritional value and quality through producing marker-assisted breeding by making crosses from promising concerned stress-tolerant genotypes and selecting among their progeny using gene-specific markers (Hika et al., 2015a; Hika et al., 2015b; Teklu et al., 2021).

Enhancement of the efficiency of the breeding strategy for Sesame using a molecular marker to investigate the phylogenetic relationship and genetic diversity is very significant (Wang et al., 2012; Park et al., 2014). Molecular markers have been widely used to check the identity and purity of cultivars and assess their genetic variability in different crops. Also, environmental factors do not influence them, and DNA can be analyzed from any growth stage (Dar et al., 2019b; Abd El-Moneim et al., 2021). Several marker systems have been utilized to investigate the genetic variability and phylogenetic associations between sesame cultivars, including Random Amplified Polymorphic DNA (RAPD) (Pham et al., 2009; Dar et al., 2017); inter simple sequence repeats (ISSR) (Nyongesa et al., 2013; Abate et al., 2015), simple sequence repeats (SSRs) (Uncu et al., 2015; Dossa et al., 2016; Sehr et al., 2016) amplified fragment length polymorphism (AFLP) (Laurentin and Karlovsky, 2007); and Sequence Related Amplified Polymorphism (SRAP) ((Ali AL-somain et al., 2017).

SCoT (Start Codon Targeted) is a strategy for creating a plant DNA marker system based on conserved sequences flanking the ATG regions of the start codon in plant genes (Collard and Mackill, 2009). No prior knowledge of the sequence under investigation is required to use this marker system. To the best of our knowledge, using SCoT marker to characterize genetic diversity in sesame germplasm has not yet been described in other molecular investigations in *Sesamum indicum* L except in the report of Maini and Dasgupta (Bhattacharjee and Dasgupta, 2020). ISSR and SCoT markers have been proven valuable in genetic diversity investigations because of their high reproducibility and great ability to detect polymorphism (Thakur et al., 2021a; Igwe et al., 2022).

Genetic drift, mutation, and natural selection within and among populations stimulate genetic diversity and differentiation of populations. Population genetics is helpful for the evolution of organisms that respond to different biotic and abiotic stresses determining resources, genetic composition, and differentiation of populations (Hancock et al., 2021).

Recently, biochemical markers to estimate genetic diversity has received much interest. Because of its simplicity and efficacy in determining crop germplasm genetic diversity, sodium dodecyl sulfate-polyacrylamide gel electrophoresis (SDS- PAGE) is commonly utilized among biochemical techniques (Nisar et al., 2011; Sharma and Krishna, 2017; Gowayed and Abd El-Moneim, 2021). Akbar *et al.* (Akbar et al., 2012) used SDS-PAGE to analyze the genetic diversity of 105 sesame accessions obtained from various agroecological locations in Pakistan. A total of 20 polypeptide bands were found, with molecular weights ranging from 13.5 to 100 kDa, 14 and 6 of which were polymorphic and monomorphic, respectively.

The most important aims in cytogenetic investigations are chromosome identification and karyotype study. In the cytological examination, karyotype study is vital for demonstrating origin, polidy, chromosome attributes, and taxonomic and phylogenetic relationships between plants (Eroğlu et al., 2013; Akbar et al., 2020; EL-Mansy et al., 2021). Karyoevolutionary forms are illustrated and demonstrated by studying karyotype attributes and their formula (Weiss-Schneeweiss et al., 2013; Soliman et al., 2019; Soliman et al., 2020). Genetic diversity of related species using Karyotype analysis was used to estimate the evolution of chromosomes and delimitate different formulae patterns that are considered the main tool in the mechanism of changes in chromosomal evolution (Kamel et al., 2014; Soliman et al., 2016). There are three cytogenetic groups for Sesame based on chromosome number included 1) first group with chromosome number 2n = 26 for *Sesamum indicum, S. alatum, S. capense, S. schenckii,* and *S. malabaricum* 2) the second group with chromosome number 2n = 32 for *S. prostratum, S. laciniatum, S. angolense*, and *S. angustifolium* and 3) third group with chromosome number 2n = 64 for *S. radiatum, S. occidentale*, and *S. schinzianum*. All *Sesamum* species are self-pollination. The cross-compatibility was limited because of the difference in chromosome number between different *Sesamum* species (Carlsson et al., 2008; Kumari and Ganesamurthy, 2015). Morinaga et al. (Morinaga et al., 1929) are the first who investigate the chromosome number of the cultivated Sesame as x = 8, 13, and 2n = 26. There are not many cytogenetic and molecular investigations on *Sesamum indicum*. (Liu et al., 2018).

Molecular and cytological relationships give informative knowledge and adequate information in plant breeding strategies that help transfer desirable characters and genes from one crop to another. Thus, this paper aimed to study genetic diversity between various cultivated genotypes of *Sesamum indicum* in Saudi Arabia using physiological, biochemical, cytological analysis, and molecular attributes (SCoT & ISSR).

## Material and methods

### Plant material

Six local sesame cultivars were provided by the Ministry of Environment, Water, and Agriculture in Saudi Arabia (Table 1).

**TABLE 1:** Locations of the studied cultivars used in this study.

Sr	Location	Cultivar
G1	Al-Ahsa	Ahs-670
G2	Asser	As-1236
G3	Al-Baha	Bah-697
G4	Jizan	Jiz-511
G5	Jizan	Jiz-517
G6	Al-Baha	Bah-312

### Morpho-physiological and agronomical parameters

Plant height, harvest index (%), seed yield (Kg/ha) total plant dry matter (kg/ha) were estimated by randomly collecting five plants (86 days) for each genotype. The field experiments were conducted in the faculty of science, Arish university. The genotypes were arranged in a randomized complete block design with three replications. Each genotype was sown in a plot size of 3.6 m, consisting of three rows of 2 m in length. Spacing between rows and plants was 60 and 15 cm, respectively, resulting in 30 plants plot-1. The experiment was irrigated by 100% of the calculated crop water requirement [?]. Fertilizer in the form of Calcium superphosphate (15.5% P_2_O_5_) at a rate of 200 kg/fed and potassium sulphate (48%K_2_O) at 100 kg/fed rate was added during soil preparation. Nitrogen in the form of ammonium nitrate (33%) at a rate of 150 kg was manually side-dressed into three portions, at sowing, after thinning, and at the flowering stage. All agricultural practices were adopted as recommended for each location. In order to compare the performance of the studied sesame genotypes, ten plants/genotypes (86 days) from each plot were selected randomly for data collection. The studied traits were plant height, seed yield (Kg/ha), harvest index (%), and total plant dry matter (kg/ha). The weight of all the seeds and stover were measured by an electrical balance and converted the yield in kg ha-1. The harvest index was calculated using the following formula:

$$Harvest\;index\left(\%\right)=\frac{Seed\;yield}{Bio\log ical\;yield}\times 100$$

Where, Biological yield = Seed yield + Stover yield.

### Biochemical Parameters

The oil from seeds for each genotype was extracted in hexane on a soxhlet apparatus. Methyl esters were obtained according to the method of Anjani and Yadav (Anjani and Yadav, 2017). The organic phase was extracted with hexane and washed with water till neutral pH. The hexane was dried over anhydrous sodium sulfate and concentrated with nitrogen gas to get methyl esters. The fatty acid composition was determined using an Agilent 7890B gas chromatograph (GC) equipped with a flame ionization detector (FID) and an autosampler.

### Genomic DNA extraction and PCR conditions

The CTAB approach (Doyle, 1990; Clarke, 2009) was used to extract DNA from 2 g of young leaves obtained from 10-day-old seedling plants of each genotype investigated. The total volume of the PCR systems was 20 μL, which contained 10 μL MIX, 8 μL ddH_2_O, 1 μL primers (10 μM), and 1 μL template DNA (50 ng/μL). Twenty ISSR and twenty-five SCoT primers were selected from previous research to be employed in this research (Table 2). The marker amplification reaction was performed according to the following program: 4 min for predenaturation at 95°C for 3 min, followed by 40 cycles of denaturation at 95°C for 40 s, annealing at 44–52°C/ISSR, and 48–55°C/SCoT for 40 s, and extension at 72°C for 2 min, with a final extension at 72°C for 7 min. The PCR products according to the primers for both markers were separated on a 1.5% agarose gel.

**TABLE 2:** Name of primer and their sequence used in the research.

ISSR name	Sequence	SCoT name	Sequences
ISSR 1	(AG)8T	SCoT 1	CAACAATGGCTACCACC
ISSR 2	(GA)8T	SCoT 2	CAACAATGGCTACCACG
ISSR 3	(CT)8T	SCoT 3	AAGCAATGGCTACCACC
ISSR 4	(CT)8A	SCoT 4	ACGACATGGCGACCAAC
ISSR 5	(CA)8T	SCoT 5	ACCATGGCTACCACCGA
ISSR 6	(GT)8A	SCoT 6	CACCATGGCTACCACCA
ISSR 7	(AG)8C	SCoT 7	ACCATGGCTACCACCGC
ISSR 8	(AG)8G	SCoT 8	ACGACATGGCGACCCAC
ISSR 9	(GA)8C	SCoT 9	CCATGGCTACCACCGCA
ISSR 10	(CT)8G	SCoT 10	ACGACATGGCGACCGCG
ISSR 11	(AC)8 C	SCoT 11	CAACAATGGCTACCACCC
ISSR 12	(TG)8 A	SCoT 12	ACCATGGCTACCACCGCG
ISSR 13	(AG)8 YT	SCoT 13	CACCATGGCTACCACCAG
ISSR 14	(AG)8 YA	SCoT 14	CCATGGCTACCACCGCCT
ISSR 15	(GA)8 YT	SCoT 15	CAACAATGGCTACCACGC
ISSR16	(CA)8 RG	SCoT 16	ACGACATGGCGACCATCG
ISSR 17	(GT)8 YG	SCoT 17	ACGACATGGCGACCCACA
ISSR 18	(AC)8 YT	SCoT 18	CCATGGCTACCACCGCAC
ISSR 19	(GAA)6	SCoT 19	ACGACATGGCGACCGCGA
ISSR 20	(CAG)5	SCoT 20	ACG ACA TGG CGA CCA CGC
		SCoT 21	ACC ATG GCT ACC ACC GGC
		SCoT 22	ACG ACA TGG CGA CCC ACA
		SCoT23	CAA TGG CTA CCA CTA CAG
		SCoT 24	ACA ATG GCT ACC ACT GAG
		SCoT 25	ACA ATG CTA CCA CCA AGC

### SDS-PAGE analysis

Protein banding patterns were analyzed using the Sodium Dodecyle Sulfate Polyacrylamide Gel Electrophoresis (SDS-PAGE) technique. Total protein was extracted according to Laemmli (Laemmli, 1970). Leaves of sesame samples were collected and were used for the total protein extraction. The protein content was determined according to the method of Bradford (Bradford, 1976). Furthermore, 1) or (0) refer to the presence or absence of each recorded protein band for each genotype.

### Data analysis

Every clear band of each primer was recorded as present 1) or absent (0). To evaluate the investigated primers’ informativeness, (PIC, EMR, MI, and Rp) parameters were determined for each primer according to Anderson et al. (1993), Powell et al. (1996), Prevost and Wilkinson (1999), Nagaraju et al. (2001) (Anderson et al., 1993; Powell et al., 1996; Prevost and Wilkinson, 1999; Nagaraju et al., 2001). GenAlEx software V. 6.5 was utilized to compute the mean number of alleles per loci (Na), the average number of effective alleles per loci (Ne), Shannon’s information index (I), expected heterozygosity (He), and unbiased expected heterozygosity (uHe) for every primer across the studied genotypes based on the frequency of alleles of each locus. Principle Component Analysis scatter diagram for cyto-molecular data was made based on a Dice coefficient genetic similarity matrix. In addition, ClustVis was utilized to construct heatmaps using the R Package (https://biit.cs.ut.ee/clustvis/) (Metsalu and Vilo, 2015). A cluster dendrogram of the investigated cultivars was created according to molecular attributes *via* the unweighted pair group method of averages (UPGMA) in NTSYSpc software V. 2.1. The Morpho-physiological, Agronomical and Biochemical Parameters data were analyzed using SAS Software, and mean differences among studied genotypes were calculated using Duncan test as post hoc at a significant 5% level.

### Cytological study

Twenty-five seeds from each genotype of Sesame were germinated in sterilized filter paper in Petri dishes at RT (25°C) for 3–5 days. Root tips with length (1–1.5 cm) were collected and pretreated using 8-hydroxyquinoline for 2 h at 4°C. Then root tips were washed with distilled water and then fixed in Alcohol: glacial acetic acid (3:1 v/v) for 24 h. The root tips are rinsed with DH_2_o and kept in Alcohol till use at 4 °C. The hydrolysis step occurred using 1.0 N HCl for 30 min at RT; then, root tips were stained with 2% aceto-orcein stain for 2 h. Counting chromosome numbers for 20 metaphase cells using Olympus CX40 microscope and photographed using a digital camera at X = 100. Karyotype parameters and ideogram assessments were estimated *via IdeoKar* software at: http://agri.uok.ac.ir/ideokar/index.htm; different karyotype parameters’ equations were calculated and presented in Supplementary Table S1.

## Results

### Physio-morphological parameters

**FIGURE 1:**
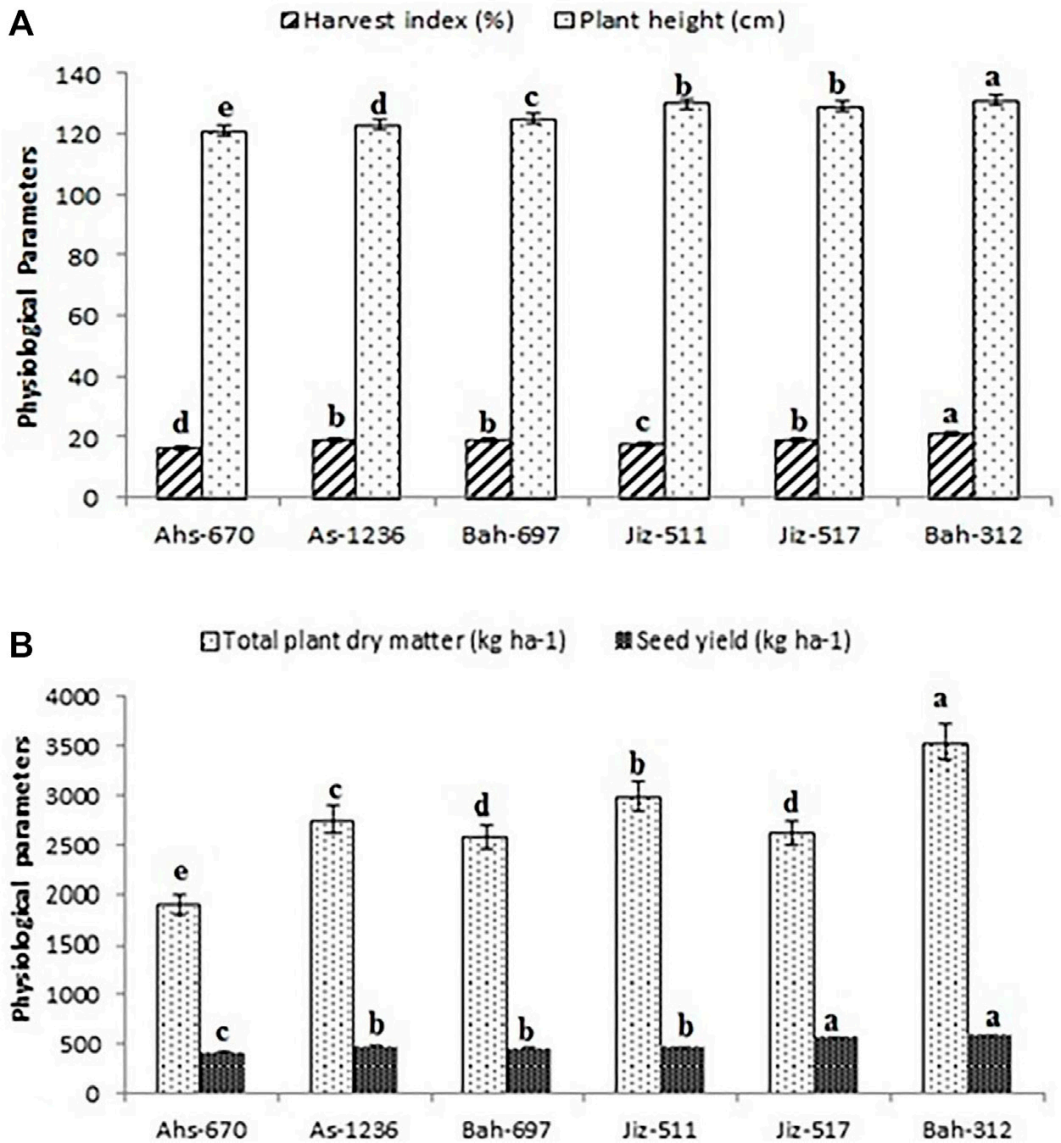
Physio-morphological parameters for different genotypes of sesame. **(A)**; harvest index (%) and Plant height (cm). **(B)**; Total plant dry matter (kg ha-1) and seed yield (kg ha-1). Values are the averages of three replicates ±SD. Different letters indicate significant differences according to Duncan’s multiple range tests (*p < 0.05*).

Genotype G6 (Bah-312) had the highest plant height, harvest index, total plant dry matter, and seed yield with values of 131 cm, 21.22 (%), 3,573.2 (Kg ha^−1^), 594.58 (Kg ha^−1^), respectively, and the lowest values were recorded in genotype G1 (Ahs-670) as shown in)Figure 1.

### Biochemical parameters

**FIGURE 2:**
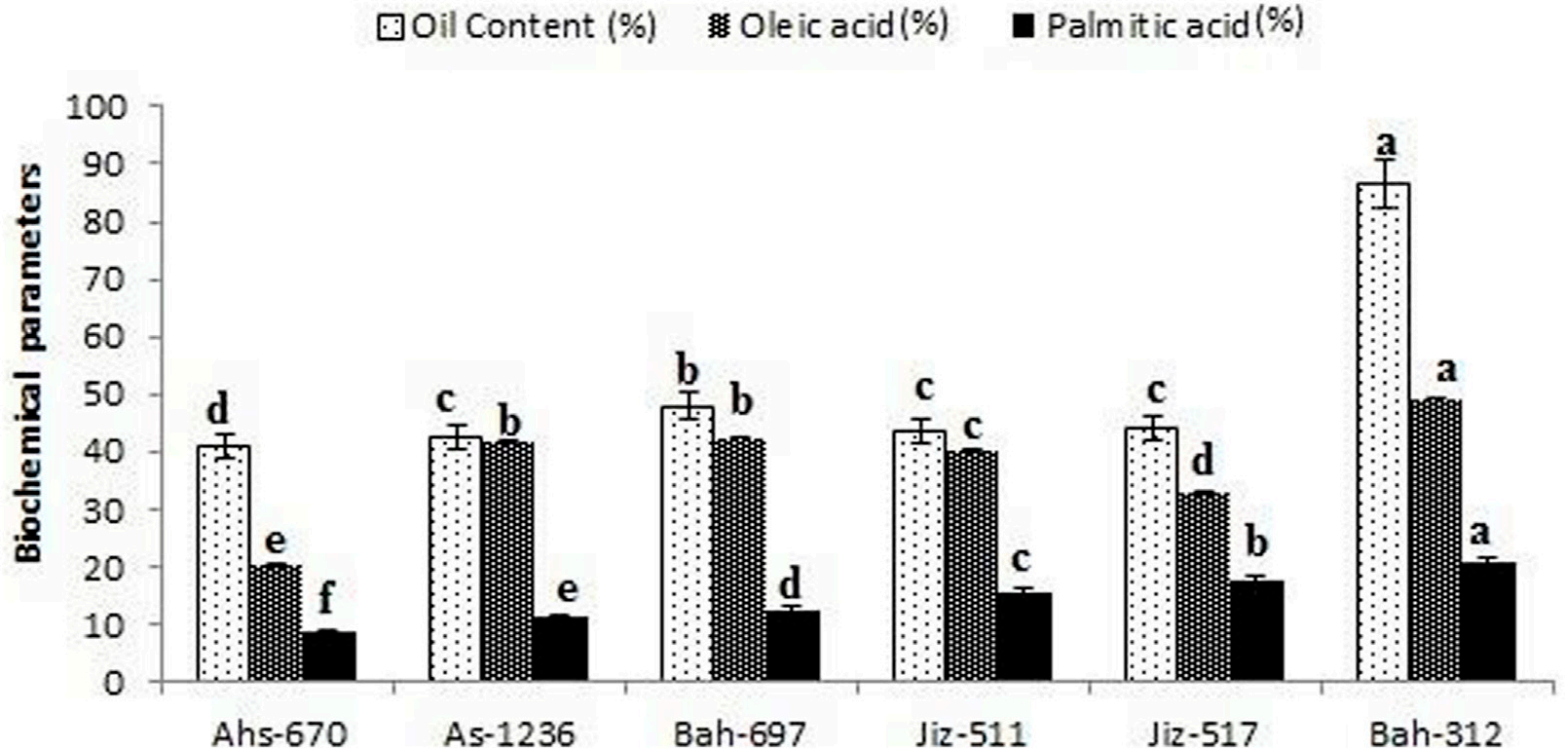
Percentage of oil content and fatty acids content (Oleic acid & Palmitic acid) for different sesame genotypes. Values are the average of three replicates ±SD. Different letters indicate significant differences according to Duncan’s multiple range tests (*p < 0.05*).

The highest values of oil content and fatty acids content (Oleic acid, Linoleic acid & Palmitic acid) were recorded in genotype G6 (Bah-312) with values of 86.22% for oil content, 49.34% for oleic acid, and 19.32% for palmitic acid as shown in)Figure 2.

### Polymorphism as revealed by ISSRs

The size of generating alleles for 20 ISSR primers varied from 75 to 1,425 bp. A total of 233 alleles were amplified in the studied samples of different genotypes used in current investigation (Table 3). Out of which, 161 were polymorphic alleles, and 72 alleles were monomorphic. The number of polymorphic alleles varied from 1 (ISSR 4) to 19 (ISSR 2), with a mean of 8.5. The average polymorphic percentage (P%) was 65.32% across all the studied genotypes. The highestP% was100% for ISSR 2,9,10, and the lowest was 16.67% for ISSR 4. Moreover, the PIC values ranged from 0.09 (ISSR 4) to 0.82 (ISSR 2), with an average of 0.49. The highest value of MI was recorded by ISSR 2 (15.50), while the least was for primer ISSR 4 (0.02), with the average 4.05. EMR values varied from 0.17 to 19 for primers ISSR four and ISSR 2, respectively, whereas the mean value was 6.33. Rp values varied from 7.01 (ISSR 2) to 21.11 (ISSR 12), whereas the mean value was 11.19 distinguishing the different genotypes. Generally, ISSR two and ISSR four primers revealed the highest and least values for P%, PIC, MI, and EMR%, respectively.

**TABLE 3:** Types and number of generated DNA bands, polymorphism percentage, and informativeness comparison obtained *via* ISSR primers.

	Number of fragments				FS bp						
	MB	UB	PB	TAB	Larger	Smaller	PIC	EMR	MI	P (%)	Rp
ISSR 1	6.00	3.00	8.00	14.00	960	180	0.41	4.57	1.89	57.14	17.22
ISSR 2	0.00	9.00	19.00	19.00	1,140	75	0.82	19.00	15.50	100.00	7.01
ISSR 3	2.00	0.00	4.00	6.00	950	445	0.39	2.67	1.05	66.67	7.28
ISSR 4	5.00	0.00	1.00	6.00	980	275	0.09	0.17	0.02	16.67	10.89
ISSR 5	1.00	6.00	12.00	13.00	1,260	370	0.75	11.08	8.31	92.31	6.50
ISSR 6	1.00	8.00	13.00	14.00	1,280	240	0.73	12.07	8.84	92.86	7.50
ISSR 7	4.00	3.00	9.00	13.00	1,330	410	0.45	6.23	2.82	69.23	14.22
ISSR 8	5.00	2.00	6.00	11.00	1,070	290	0.47	3.27	1.54	54.55	11.67
ISSR 9	0.00	3.00	7.00	7.00	1,540	330	0.81	7.00	5.67	100.00	2.67
ISSR 10	0.00	6.00	17.00	17.00	1,245	160	0.75	17.00	12.83	100.00	8.33
ISSR 11	7.00	2.00	2.00	9.00	1,425	260	0.22	0.44	0.10	22.22	14.11
ISSR 12	10.00	2.00	4.00	14.00	1,330	215	0.25	1.14	0.28	28.57	21.11
ISSR 13	4.00	3.00	10.00	14.00	1,370	190	0.51	7.14	3.66	71.43	13.67
ISSR 14	2.00	4.00	11.00	13.00	1,280	290	0.57	9.31	5.33	84.62	11.11
ISSR 15	1.00	3.00	12.00	13.00	1,040	210	0.62	11.08	6.91	92.31	9.77
ISSR 16	6.00	0.00	4.00	10.00	1,400	125	0.29	1.60	0.47	40.00	14.11
ISSR 17	6.00	0.00	3.00	9.00	980	230	0.22	1.00	0.22	33.33	14.00
ISSR 18	3.00	4.00	7.00	10.00	380	95	0.51	4.90	2.48	70.00	9.89
ISSR 19	4.00	2.00	6.00	10.00	1,110	240	0.47	3.60	1.69	60.00	10.61
ISSR 20	5.00	2.00	6.00	11.00	1,065	280	0.44	3.27	1.45	54.55	12.22
Total	72.00	62.00	161.00	233.00			9.79	126.54	81.05	69.1%	223.90
Average	3.60	3.10	8.05	11.65			0.49	6.33	4.05	65.32	11.19

MB, monomorphic band; UB, unique band; PB, polymorphic band; TAB, total amplified bands; FS, fragment size; PIC, polymorphic information content; EMR, effective multiplex ratio; MI, marker index; P%, percent of polymorphism; Rp, resolving power.

### Polymorphism as revealed by SCoT

As shown in Table 4, out of 275 amplified alleles, 214 and 76 alleles were polymorphic and monomorphic, respectively, when studying 25 SCoT primers. The size of generated alleles ranged from 80 to 2,150 bp. Furthermore, SCoT 21 & three primers had the highest 31) and lowest 2) number of polymorphic alleles. The average P% was 77.8% across all genotypes. The highest P% was (100%) for primers SCoT 4, 12, 14, 21, and 25, while the least was 33.33% for primers SCoT three and 5. The highest value for PIC was 0.92 for primer SCoT 25. While the lowest value was 0.10 for primer SCoT 3, and the mean was 0.55/primer. On the other hand, the highest (27.16) and lowest values (0.07) of MI were observed for SCoT 21 and three respectively, and the average value was 4.93. Furthermore, the values of EMR varied from 0.67 to 31 for SCoT 3 and 21 primers, respectively, and the average was 7.16. The Rp values varied between 2.83 (SCoT 6) to 19.72 (SCoT 7). While the average Rp was 8.92. In conclusion, primers SCoT 21 and three revealed the highest and least values for %P, PIC, MI, and EMR, respectively.

**TABLE 4:** Types and number of generated DNA bands, polymorphism percentage, and informativeness comparison obtained *via* SCoT primers.

	Number of fragments				FS bp						
	MB	UB	PB	TAB	Larger	Smaller	PIC	EMR	MI	P (%)	Rp
SCoT 1	4.00	1.00	5.00	9.00	885	260	0.27	2.78	0.75	55.56	13.11
SCoT 2	2.00	0.00	8.00	10.00	1,480	285	0.39	6.40	2.49	80.00	12.22
SCoT 3	4.00	0.00	2.00	6.00	480	150	0.10	0.67	0.07	33.33	10.78
SCoT 4	0.00	5.00	13.00	13.00	1,700	445	0.84	13.00	10.89	100.00	4.22
SCoT 5	6.00	0.00	3.00	9.00	1,520	350	0.16	1.00	0.16	33.33	15.17
SCoT 6	1.00	6.00	7.00	8.00	2,150	500	0.82	6.13	5.04	87.50	2.83
SCoT 7	8.00	1.00	5.00	13.00	1,430	330	0.24	1.92	0.46	38.46	19.72
SCoT 8	2.00	2.00	7.00	9.00	530	350	0.51	5.44	2.79	77.78	8.78
SCoT 9	4.00	1.00	5.00	9.00	1,250	470	0.32	2.78	0.89	55.56	12.22
SCoT 10	1.00	2.00	4.00	5.00	1,580	830	0.61	3.20	1.95	80.00	3.89
SCoT 11	2.00	5.00	8.00	10.00	1,210	350	0.69	6.40	4.44	80.00	6.11
SCoT 12	0.00	1.00	12.00	12.00	1,370	200	0.67	12.00	8.03	100.00	7.95
SCoT 13	2.00	2.00	6.00	8.00	1,530	340	0.63	4.50	2.83	75.00	5.94
SCoT 14	0.00	4.00	10.00	10.00	950	280	0.71	10.00	7.14	100.00	5.72
SCoT 15	1.00	2.00	8.00	9.00	2040	510	0.54	7.11	3.84	88.89	8.28
SCoT 16	6.00	1.00	4.00	10.00	380	80	0.35	1.60	0.56	40.00	13.00
SCoT 17	2.00	1.00	7.00	9.00	1,490	310	0.56	5.44	3.06	77.78	7.89
SCoT 18	2.00	0.00	4.00	6.00	200	100	0.54	2.67	1.43	66.67	5.56
SCoT 19	2.00	1.00	9.00	11.00	270	100	0.69	7.36	5.10	81.82	6.78
SCoT 20	2.00	2.00	9.00	11.00	330	100	0.65	7.36	4.76	81.82	7.78
SCoT 21	0.00	18.00	31.00	31.00	1940	90	0.88	31.00	27.16	100.00	7.67
SCoT 22	5.00	5.00	9.00	14.00	1,400	200	0.53	5.79	3.06	64.29	13.17
SCoT 23	1.00	3.00	13.00	14.00	930	110	0.62	12.07	7.45	92.86	10.72
SCoT 24	4.00	2.00	7.00	11.00	420	170	0.53	4.45	2.34	63.64	10.44
SCoT 25	0.00	11.00	18.00	18.00	1,300	155	0.92	18.00	16.50	100.00	3.00
Total	61.00	76.00	214.00	275.00			13.76	179.08	123.20	77.8%	222.95
Average	2.44	3.04	8.56	11.00			0.55	7.16	4.93	74.17	8.92

MB, monomorphic band; UB, unique band; PB, polymorphic band; TAB, total amplified bands; FS, fragment size; PIC, polymorphic information content; EMR, effective multiplex ratio; MI, marker index; P%, percent of polymorphism; Rp, resolving power.

### Positive and negative specific markers revealed by ISSR and SCoT

**FIGURE 3:**
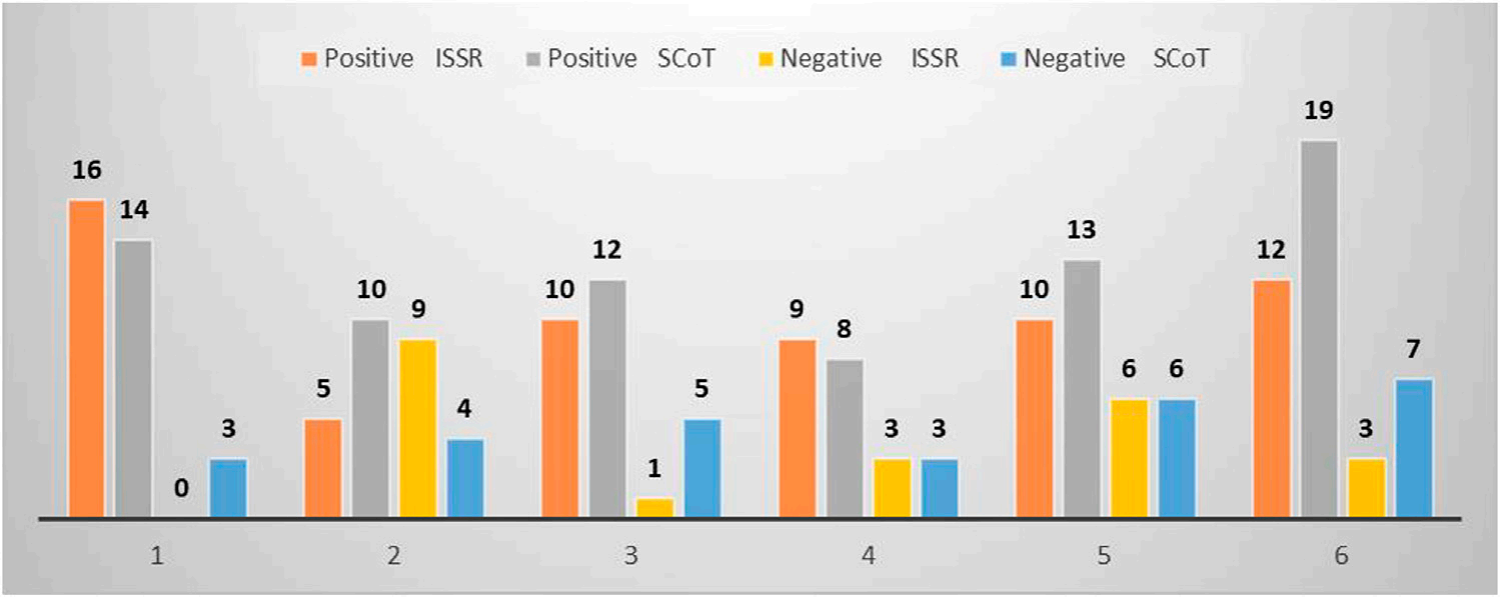
Total number of positive and negative unique bands revealed by ISSR and SCoT of the studied cultivars. (1) Ahs-670, (2) As-1236, (3) Bah-697, (4) Jiz-511, (5) Jiz-517 and (6) Bah-312

The number of genotype-specific markers (positive and negative) scored across studied genotypes was as high as 188, in which 84 of them were generated from ISSR, while 104 were from SCoT analysis (Figure 3). However, ISSR two showed the highest unique bands (nine markers) while SCoT 21 exhibited the highest number of unique bands (18 markers). The highest number of unique bands across both types of markers was exhibited by genotype 6 (41 amplicons), 15 markers generated by ISSR, and 26 markers amplified by SCoT, while the least was scored for genotype 4 (23 amplicons).

### Polymorphism as revealed by SDS-PAGE analysis

**FIGURE 4:**
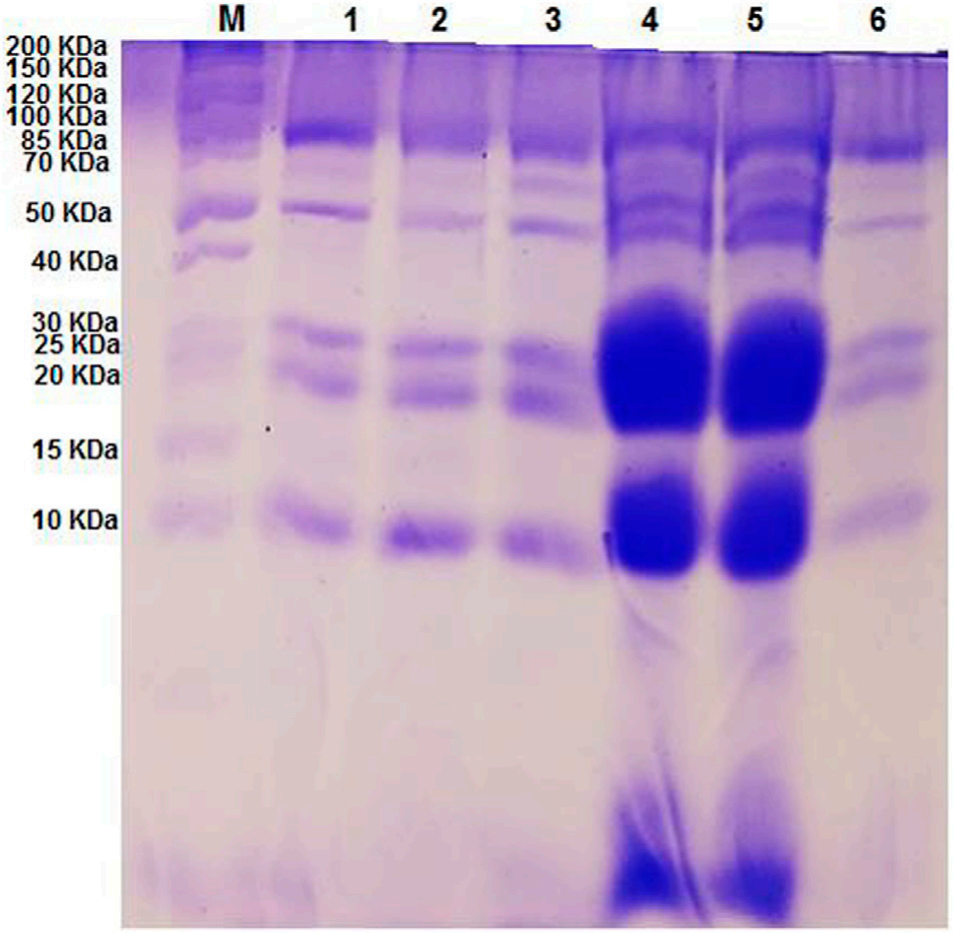
SDS-PAGE protein profile of studied genotypes. (M) = marker protein) (1) Ahs-670, (2) As-1236, (3) Bah-697, (4) Jiz-511, (5) Jiz-517 and (6) Bah-312

Eight polypeptides’ bands were scored amongst the six sesame genotypes. Of these eight bands, 4 (50%) were polymorphic, and 4 (50%) were monomorphic. The size of the protein bands amplified by SDS-PAGE ranged from 2 to 85 kDa. Bands 10, 20, 50, and 85 were present in all the genotypes, whereas bands 2 and 59 kDa were present only in G4 and G5. Table 5 concluded that G4 and G5 scored the highest amplified bands (seven). In addition, the highest polymorphism percentage was 43% for G4 and G5 genotypes. Meanwhile, the least polymorphism percentage was 20% for G1 and G2 genotypes. Additionally, no positive or negative specific bands amplified among the studied genotypes were observed. The variability in the intensity of the studied genotypes was viewed in G4 and G5 protein bands that exhibited the amount of protein peptides increasing at a specific molecular weight (Figure 4).

**TABLE 5:** Number and types of amplified protein bands and polymorphism percentages.

	TAB	MB	PB	UB	%P (%)
G1	5	4	1	0	20
G2	5	4	1	0	20
G3	6	4	2	0	33.33
G4	7	4	3	0	42.86
G5	7	4	3	0	42.86
G6	6	4	2	0	33.33

TAB, total amplified bands; MB, monomorphic band; PB, polymorphic band; UB, unique band, %P percent of polymorphism.

### Phylogenetic relationship as revealed by cluster analysis using SDS-PAGE, ISSR, and SCoT data

**FIGURE 5:**
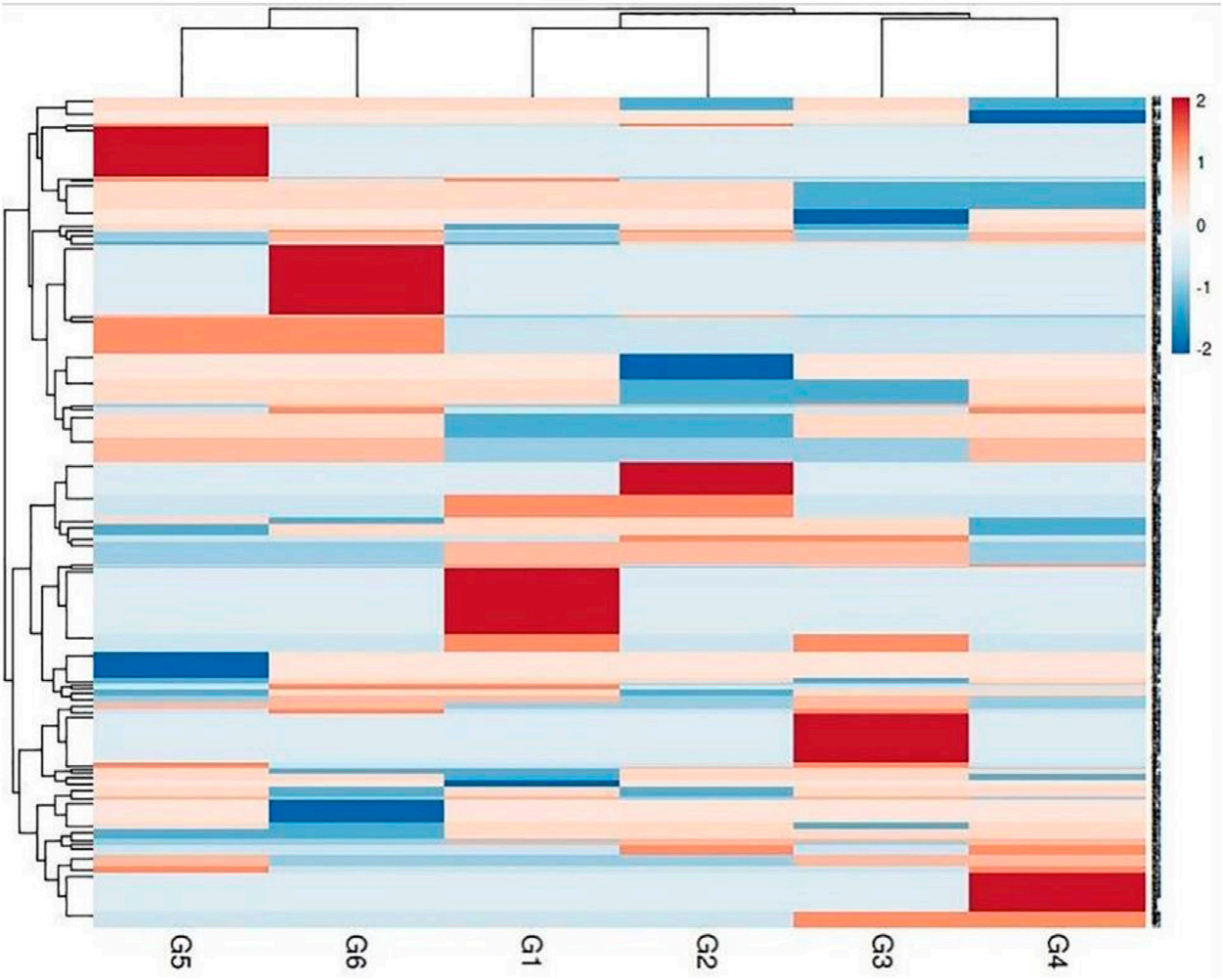
Multivariate heatmap illustrating the genetic diversity of studied sesame cultivars, based on the 20 ISSR, 25 SCoT primers, and SDS-PAGE profile using the module of a heatmap of ClustVis—an online tool for clustering and visualizing multivariate data. (1) Ahs-670, (2) As-1236, (3) Bah-697, (4) Jiz-511, (5) Jiz-517 and (6) Bah-312

The genetic variance between the studied genotypes was detailed in the multivariate similarity heatmap in (Figure 5). The multivariate similarities heatmap was conducted using R Package based on SDS-PAGE, ISSR, and SCoT data; the six genotypes were clustered into two main clades. The first clade contained two sub-clades; the first subclade contained genotypes G3& G4, and the second sub-clade contained genotypes (G1 & G2). The second clade contained genotypes (G5 & G6).

### Population genetics and genetic diversity

A total of 20 ISSR primers and 25 SCoT primers were used to study the population genetics of the Sesame genotypes; the genetic diversity data was illustrated in Table 6. For the ISSR marker, the number of different alleles (Na) per locus varied from 1.261 in G5 to 1.359 in G6; the number of effective alleles (Ne) per locus varied from 1.308 in G3 to 1.333 in G6. The Shannon’s index (I) varied from 0.260 in G1 to 0.318 in G6; the expected heterozygosity (He) values ranged from 0.161 in G1 to 0.218 in G6, where Unbiased Expected Heterozygosity (uHe) values varied from 0.231 in G1 to 0.290 in G6. Regarding the SCoT marker, the number of different alleles (Na) per locus varied from 1.141 in G1 to 1.310 in G6; the number of effective alleles (Ne) per locus varied from 1.260 in G1 to 1.357 in G6. The Shannon’s index (I) was varied from 0.223 in G1 to 0.306 in G6; the expected heterozygosity (He) values ranged from 0.153 in G1 to 0.209 in G6, where Unbiased Expected Heterozygosity (uHe) values varied from 0.203 in G1 to 0.279 in G6; the percentage of polymorphism was varied from43.19% in G6 to 52.56% in G1 for ISSR marker, where polymorphism percentage ranged from 36.82% in G6 to 50.54% in G1 for SCoT marker. In general, the highest value of genetic diversity (He) was 0.195 for the ISSR marker.

**TABLE 6:** Genetic parameters for the studied Sesame genotypes using 20 ISSR primers and 25 SCoT primers.

Genotypes	ISSR					SCoT				
	Na	Ne	I	He	uHe	Na	Ne	I	He	uHe
Ahs-670	1.291	1.318	0.260	0.161	0.231	1.141	1.260	0.223	0.153	0.203
As-1236	1.303	1.363	0.310	0.212	0.283	1.249	1.340	0.290	0.199	0.265
Bah-697	1.265	1.308	0.264	0.181	0.241	1.224	1.304	0.260	0.178	0.237
Jiz-511	1.282	1.332	0.284	0.195	0.260	1.238	1.324	0.277	0.190	0.253
Jiz-517	1.261	1.314	0.269	0.184	0.245	1.144	1.265	0.227	0.156	0.207
Bah-312	1.359	1.372	0.318	0.218	0.290	1.310	1.357	0.306	0.209	0.279
Total	1.293	1.333	0.285	0.195	0.260	1.218	1.308	0.264	0.181	0.241

Na, No of Different Alleles; Ne, No of Effective Alleles = 1/(*p*^2 + *q*^2); I, Shannon’s Information Index = −1* [*p* * Ln (*p*) + *q* * Ln(q)]; He, Expected Heterozygosity = 2 *p *q; uHe, Unbiased Expected Heterozygosity = [2N/(2N-1)] * He; q, (1–Band Freq.) ^0.5 and *p* = 1–q.

### Cytological analysis

**FIGURE 6:**
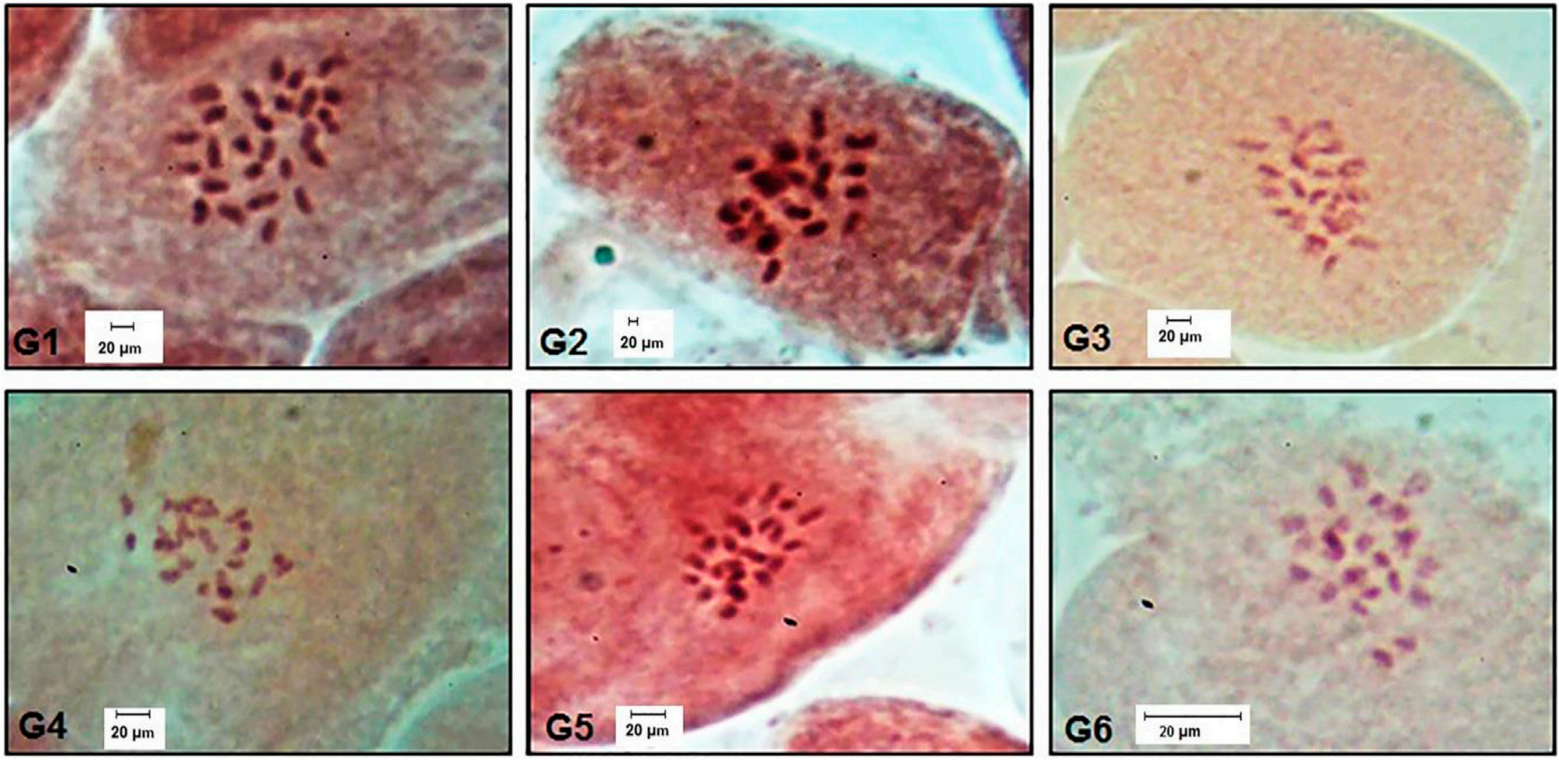
Chromosome number for all studied genotypes 2n = 26. (G1) Ahs-670, (G2) As-1236, (G3) Bah-697, (G4) Jiz-511, (G5) Jiz-517 and (G6) Bah-312.

**FIGURE 7:**
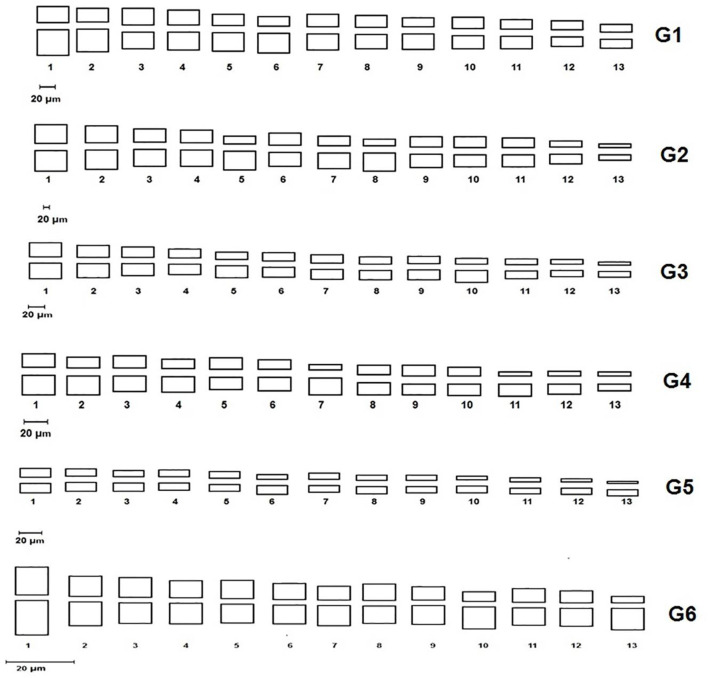
Ideogram for haploid chromosome number of the studied Sesame genotypes. (G1) Ahs-670, (G2) As-1236, (G3) Bah-697, (G4) Jiz-511, (G5) Jiz-517 and (G6) Bah-312.

**FIGURE 8:**
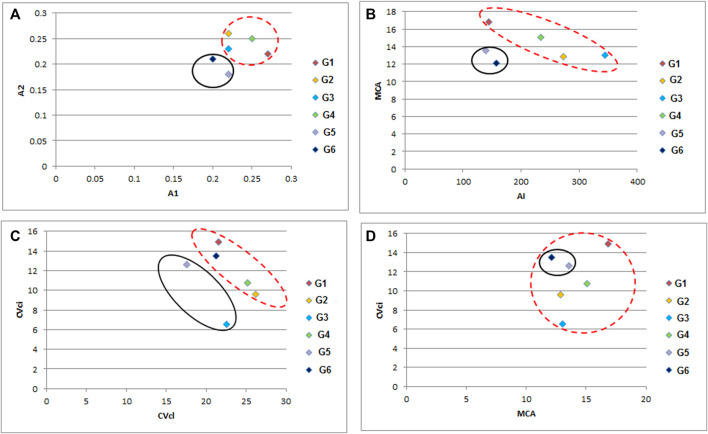
A scattered diagram of different karyotype asymmetry between different six sesame genotypes **(A)** A1 *versus* A2 parameter, **(B)** MCA *versus* AI parameter, **(C)** CVcl *versus* CVci parameter, and **(D)** MCA *versus* CVci parameter. (G1) Ahs-670, (G2) As-1236, (G3) Bah-697, (G4) Jiz-511, (G5) Jiz-517 and (G6) Bah-312.

All studied genotypes from Sesame with chromosome number 2n = 26 as shown in (Figure 6); karyotype attributes differed between different genotypes as shown in Table 7, and the ideogram for the haploid chromosome number was illustrated for the studied genotypes in (Figure 7). Haploid chromosome length varied from the lowest value 88.76 µ in genotype G3 to the highest value 119.09 µ in genotype G6. Genotype (G) has the highest values from karyotype asymmetry index (AsK%), the degree of asymmetry of karyotype (A), mean centromeric asymmetry (McA), coefficient of variation of the centromeric index (CVci), and intrachromosomal asymmetry index (A1) with value 58.50, 0.17, 16.83, 14.92 and 0.27 respectively. The highest percentage from total form percentage (TF%), centromeric index (CI) with values 44.06% and 0.44 respectively, was recorded in genotype G6; also, G6 presented the lowest value from A and A1 0.12 and 0.20, respectively. Stebbins formula was 2B in all genotypes except genotype G4 was 1B, and G5 was 2A. Karyotype formula illustrated nearly submetacentric (-), nearly metacentric (nm), and nearly subtelocentric (nst) in all genotypes. The scatter diagrams of different karyotype parameters between the studied six genotypes are illustrated in (Figure 8). The Scatter diagrams were conducted to assess the classification strength and demonstrate the relationship among different genotypes. Panels (a, b and c) between A1 and A2, between MCA and AI, and between Cvci and Cvcl clustered the genotypes into two groups: genotypes (G5 & G6) in separate groups and genotypes (G1-G4) in another group. Where scatter diagram between MCA and Cvci (Panel d) recorded that genotypes (G5& G6) are interposed inside the second groups containing the other four genotypes.

**TABLE 7:** Different Karyotype parameters and Karyotype formula for the studied Sesame genotypes.

Genotypes	Chromosome number (2n)	HCL (µ)	TF%	AsK%	S%	CI	A	McA	CVcl	CVci	AI	Stebbins	A1	A2	KF
G1	26	108.47	41.50	58.50	43.24	0.42	0.17	16.83	21.54	14.92	144.33	2B	0.27	0.22	2 nsm (−)+12 nst+12 nm
G2	26	127.33	43.82	56.18	28.06	0.43	0.13	12.86	26.19	9.60	272.83	2B	0.22	0.26	2 nsm (−)+8 nst+14 nm
G3	26	88.76	43.77	56.23	34.95	0.43	0.13	13.03	22.55	6.54	344.63	2B	0.22	0.23	4 nsm (−)+10 nst+12 nm
G4	26	103.85	42.25	57.75	40.68	0.43	0.15	15.07	25.17	10.76	233.96	1B	0.25	0.25	6nsm (−)+8 nst+10 nm
G5	26	115.40	43.47	56.53	51.28	0.43	0.14	13.56	17.56	12.62	139.22	2A	0.22	0.18	4nsm (−)+10 nst+12 nm
G6	26	119.09	44.06	55.94	41.43	0.44	0.12	12.11	21.24	13.50	157.36	2B	0.20	0.21	8 nsm (−)+10 nst +8 nm

THL, total haploid chromosome length; TF%, total form percentage; ASK, karyotype asymmetry index; S%, karyotype symmetry; CI, centromeric index; A, The degree of asymmetry of karyotype; MCA, mean centromeric asymmetry; CVCL, coefficient of variation of chromosome length; CVCI, coefficient of variation of centromeric index; AI, asymmetry index; A1, intrachromosomal asymmetry index; A2, interchromosomal asymmetry index; nsm, nearly submetacentric; nst, nearly subtelocentric; nm, nearly metacentric*.*

### Combined cytological, biochemical (SDS-PAGE), and molecular markers (ISSR &SCoT) analysis

**FIGURE 9:**
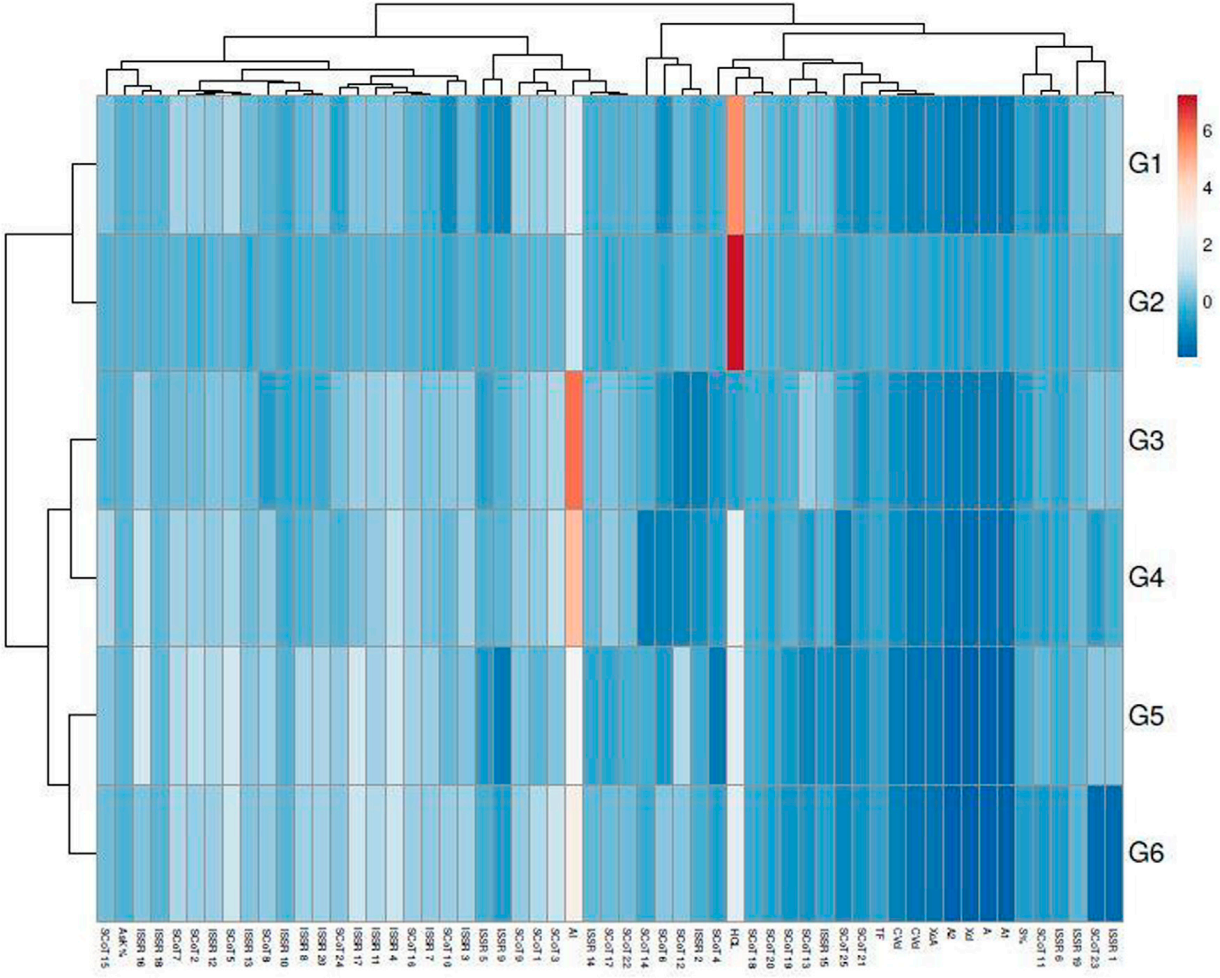
Cluster tree for the studied six sesame genotypes based on all molecular markers and cytological parameters. (G1) Ahs-670, (G2) As-1236, (G3) Bah-697, (G4) Jiz-511, (G5) Jiz-517 and (G6) Bah-312.

**FIGURE 10:**
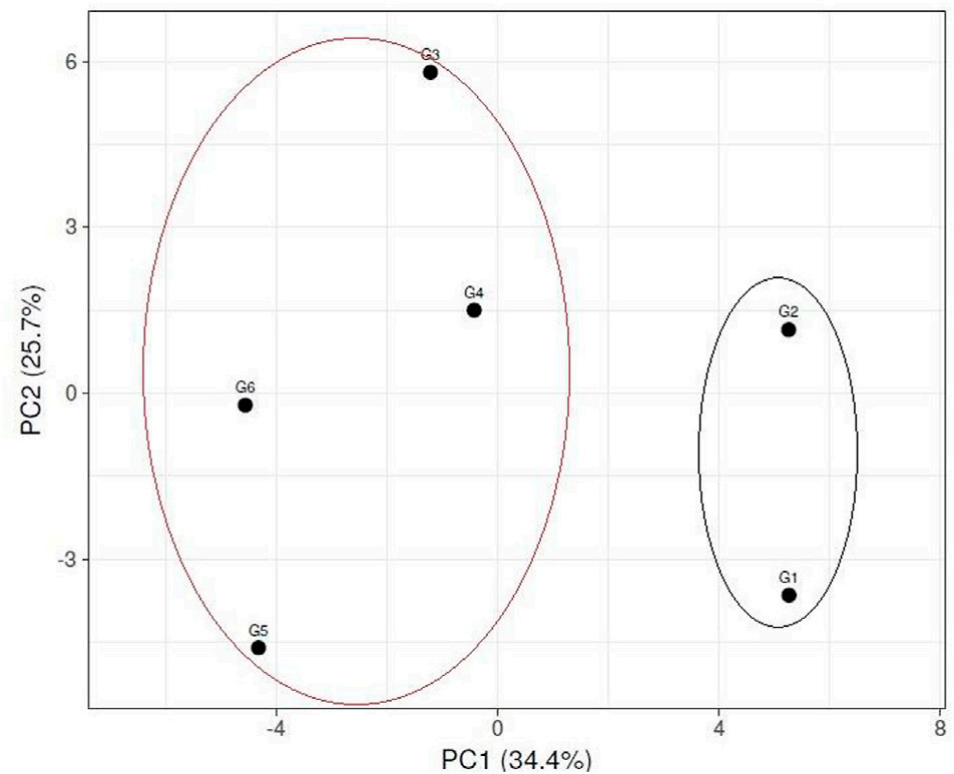
Principle Component Analysis (PCA) illustrated the genetic diversity between six sesame genotypes using molecular and cytological data. (G1) Ahs-670, (G2) As-1236, (G3) Bah-697, (G4) Jiz-511, (G5) Jiz-517 and (G6) Bah-312.

Multivariate similarity heatmap analysis using cytological, biochemical (SDS-PAGE), and molecular markers (ISSR &SCoT) was utilized to illustrate the genetic distance between sesame genotypes and illustrated the classification of these genotypes in (Figure 9). The sesame cultivars were grouped into two main clusters; the first cluster contained two sub-cluster: the first sub-cluster involved genotypes G5 and G6, the second sub-cluster included genotypes G3 and G4. The second cluster contained genotypes G1 and G2. Principle component analysis is a multivariate analysis for data used to visualize relationships, similarities, and dissimilarities among various cyto-molecular data against different sesame genotypes. Principle component analysis (PCA) for all cyto-molecular data for the six sesame genotypes was illustrated in (Figure 10). PCA explained the maximum variation interaction by cyto-molecular attributes ordinated the studied genotypes into two groups; one group contained genotypes G1 and G2, and the second group contained genotypes (G1-G4) in a separate one. The first two principal components (PC1 & PC2) with total variance 34.4% and 25.7% respectively. These values are considered the best measure for the quality of ordination and, the strength of the genotypes–morpho‐physiological relationship.

## Discussion

The significant genetic variation in *Sesamum indicum* L. should be considered when developing conservation strategies and breeding programs. Morphological and molecular analyses confirmed this. Identification based on morphological markers usually requires much time and tedious work (Abd El-Moneim et al., 2021). Furthermore, characterization of genetic variability in sesame germplasm utilizing molecular markers is minimal. Likewise, single neutral molecular marker technology has limitations in studying Sesame’s genetic diversity and relationships in-depth. Different neutral molecular marker technologies target different regions. Thus, the extensive use of neutral molecular markers targeting different regions is conducive to the comprehensive elucidation of plant genetic diversity and relationships (Gogoi et al., 2020). To date, many applications combining multiple molecular marker techniques have been employed to evaluate the genetic diversity and genetic relationships of plants (Antony et al., 2015; Thakur et al., 2021b; Tikendra et al., 2021). The combination of 45 ISSR and SCoT primers produced a higher number of alleles (508) with an average of 69% and 78% (P), demonstrating the usefulness of the selected primers for identifying the genetic variability between the studied cultivars.

The use of many polymorphic ISSR primers is of great utility as it raises the accuracy of the explanation of the results, especially if the amplified profiles are reproducible (Handaji et al., 2012). Our results revealed 100% polymorphism generated by different primers (ISSR 2,9,10) and (SCoT 4, 12,14,21, and 25) that is possibly be caused by the genetic material examined, the nature of the ISSR primers, and the hybridization temperatures utilized (Sánchez de la Hoz et al., 1996). In the same context, the average polymorphism rate (69.1%) was higher than that stated in earlier research using ISSR markers to explore the genetic diversity in Indian Sesame (57%) (Kumar and Sharma, 2011) and Korean Sesame (33%) (Kim et al., 2002). In contrast, it was lower than that recorded in Sesame from Africa and wild relatives (70.6%) (Nyongesa et al., 2013), Ethiopian Sesame (75.86%) (Woldesenbet et al., 2015), and by Anitha *et al.* (Anitha et al., 2010) (98.5%), (El Harfi et al., 2021), (80.7%). Our findings concluded that ISSR-PCR analysis is helpful for the identification of germplasms and assessment of genetic diversity among the sesame genotypes. These findings are in accordance with the previous study of (Pradeep Reddy et al., 2002; Sharma et al., 2009; Abate et al., 2015), who reported that ISSR markers are a proper method in identifying high genetic diversity in sesame germplasm. A limited number of research exists on using SCoT markers to evaluate the genetic diversity between sesame genotypes. In this regard, Bhattacharjee and Dasgupta, (2020) used EST-SSR and SCoT markers to find out the genetic diversity between 30 sesame cultivars. The size of SCoT markers ranged between 200 and 1,500 bp, and alleles mean it was 9.6 alleles/primer, while in our study ranged between 80 and 2,150 bp with alleles mean 11 alleles/primer. The discriminatory power of primers is measured by the polymorphism Information Content (PIC) value, which is utilized as a relative measure of polymorphism level. In other words, PIC is used in linkage studies to determine the informativeness of a genetic marker. The closer a primer’s value gets to 1, the more polymorphic it is and the more likely it is to reveal allelic variation and *vice versa*. The majority of primers utilized produced polymorphic profiles with varying levels of polymorphism information. The lowest PIC values were generated by (ISSR four and SCoT 3), indicating the lowest diversity of these primers. In contrast, the highest values were found in (ISSR two and SCoT 25) primers which give 100% polymorphic bands, proving that it has the best ability to discriminate among studied cultivars. The average of PIC was 0.49 & 0.55 for ISSR and SCoT, respectively, suggesting that both markers are informative and useful for distinguishing the studied genotypes. These values are lower than those obtained by (Kesawat et al., 2015) (PIC = 0.675) using the ISSR marker and (Bhattacharjee and Dasgupta, 2020) (PIC = 0.79) using SCoT markers. Oppositely, the findings of PIC values in this research exceeded those of (El Harfi et al., 2021) (PIC = 0.169) and (Zhang et al., 2012), who showed an average of 0.20 in minicore and 0.18 in the Chinese sesame core collection.

Besides PIC, we assessed the discriminatory power of the studied primers *via* calculating the MI (marker index), which is a feature of a marker, EMR (effective multiplex ratio) that is the result of the fraction of polymorphic bands and the number of polymorphic bands. Consequently, the higher polymorphism reveals a higher EMR. In addition, the ability of a primer/marker combination to detect changes between several genotypes is measured by its resolving power (Rp) (Prevost and Wilkinson, 1999); these characteristics have not yet been described before in other molecular research in *Sesamum indicum* L. Several investigations have studied these features to estimate the discriminatory power of molecular marker systems, e.g., Wheat (using ISSR, EMR = 11.07, MI = 7.81, Rp = 8.7) and (using SCoT, EMR = 9.37, MI = 6.71, Rp = 8.78) (Abd El-Moneim, 2020). Tomato (using ISSR, EMR = 2.33, MI = 1.03, Rp = 12.50) and (SCoT, EMR = 4.28, MI = 2.27, Rp = 14.14) (Abdein et al., 2018). Squash (using ISSR, EMR = 3.86, MI = 2.29, Rp = 6.65) and (using SCoT, EMR = 6.76, MI = 4.77, Rp = 6.76) (Abdein et al., 2021). Our study exhibited that the average EMR, MI, and Rp values was (6.33& 7.16), (4.05 & 4.93), and (11.19 &8.92) using ISSR and SCoT, respectively. These means were higher that means reported by (Dar et al., 2017) using SSR (EMR = 1, MI = 0.172, Rp = 0.213) and using RAPD, EMR = 6.034, MI = 1.426, Rp = 4.012). In contrast, our results were lower than (Laurentin and Karlovsky, 2007) using AFLP (MI = 10.34, Rp = 18.94). In general, our results showed that primers (ISSR 2 & SCoT 21) and (ISSR 4 & SCoT 3) had the highest and least values for (P%, PIC, MI, and EMR), respectively, demonstrating that these primers can be used to investigate molecular polymorphism among studied genotypes. Additionally, 188 negative and positive unique amplicons were generated overall for the studied primers. 108 amplicons were generated *via* SCoT primers, while 84 amplicons were generated *via* ISSR primers. Accordingly, the SCoT marker evaluated the genetic relationships among sesame genotypes more effectively than ISSR. As a result, it could be a helpful marker system for population genetics, genetic diversity, and genotype improvement research. These results were in harmony with (Abdein et al., 2018).

SDS-PAGE techniques are effectively employed for analyzing the genetic diversity in various species of Wheat (Gowayed and Abd El-Moneim, 2021), *Brassica* (Choudhary et al., 2015), and tomato (Hameed et al., 2014). In addition, protein types and their diversity vary between crop species, which may help in the early detection of species at the seed level and acquiring the information on clarity of genetic assets (Rahman and Hirata, 2004). Our findings exposed a limited level of diversity between the studied sesame genotypes. These findings agreed with Quenum *et al.* (Quenum and Yan, 2017), who exhibited a small level of intraspecific variability for seed protein between Sesame. Contrary, Nisar *et al.* (Nisar et al., 2007) showed a high level of intraspecific variability for seed protein between local and exotic chickpea germplasm. Furthermore, our results agreed with Akbar *et al.* (Akbar et al., 2012), who showed that sesame genotypes that exhibited a similar protein banding pattern might be duplicated; it must be verified using advanced molecular markers. In the same context, population genetics analysis of sesame genotypes was assessed using molecular markers. The mean number of effective alleles Ne = 1.333 for the ISSR marker and 1.308 for SCoT marker were lower than the effective alleles among 277 sesame accessions using 14 SSR primers accumulated from fifteen countries in four distinct continents (Park et al., 2014). The mean of expected heterozygosity was 0.195 for ISSR and 0.181 for SCoT, lower than values of 0.538, 0.30, 0.72, and 0.34 reported from (Hika et al., 2015b; Asekova et al., 2018; Araújo et al., 2019; Teklu et al., 2021) respectively using 14, 27, 23 and 10 SSR primers. The lowest genetic diversity and heterozygosity recorded in this work suggested the low genetic variation among studied genotypes. Low genetic variability indicated population fragmentation, leading to limited gene flow (Leimu et al., 2010). Maintaining diversity may be difficult because of the limited size and quantity of populations. Genetic drift, self-pollination, and inbreeding are some of the effects of small populations that cause genetic diversity to be diminished (Ferreira et al., 2013).

Additional cytogenetic analysis was carried out to examine the existing genetic diversity between the studies germplasm and validate or disprove the outcomes of the molecular marker analysis. Cytological parameters, including chromosome number, karyotype formula, and chromosome behavior between different cultivars, gave effective information on plants’ structural changes and evolution. The karyotype formula is one of the most crucial factors in identifying plant species and cultivars (Sharma and Sharma, 2014; Soliman et al., 2016). Karyotype parameters were used to evaluate the relationships among different cultivars, including the chromosome size, position of the centromere, and the number of chromosomes (Kolar et al., 2012). The chromosome number of all investigated sesame cultivars was 2n = 26, agreeing with (Raghavan and Krishnamurthy, 1947; Kobayashi, 1949; Nyongesa et al., 2014; Jha et al., 2020). The basic chromosome number was x = 8 and x = 13 (Kobayashi, 1991). There are no reports on the cytogenetics of sesame cultivars in KSA until now. This may be because the Sesame contains very small size of chromosomes, and the research on cytogenetics of Sesame was few over the world (Raghavan and Krishnamurthy, 1947; Mukherjee, 1959). Changes to an asymmetric karyotype can arise by shifts in the position of the centromere towards the telomere (intrachromosomal) and/or by the addition or deletion of chromatin from some but not all chromosomes, which leads to differences in size among the largest and smallest chromosome (interchromosomal) (Peruzzi et al., 2009). The karyotype parameters appraised the plant evolution using indices of symmetry. The value of TF% ranged from 0 to 50 according to (Zuo and Yuan, 2011), and the S % index ranged from 0 to 100 according to (Huziwara, 1962). The symmetric karyotype is well-conceived as primitive, and the asymmetric karyotype is offered with advanced characters (Stebbins, 1971). According to the highest ASK% and lowest TF% values, sesame G1 was the most advanced and G6 most primitive. Also, according to A and A1 sesame genotypes, G1 was the most asymmetrical, and G6 was the most symmetrical genotype (Zarco, 1986; EL-Mansy et al., 2021). Cytological data revealed that genotype G6 was the most symmetrical than others, which may be due to this genotype having the highest number of unique positive bands 19) generated from the SCoT marker. At the same time, genotype G1 was the most asymmetrical, maybe due to having the highest number of unique positive bands 16) generated from ISSR marker.

## Conclusion

Genetic diversity is required to evaluate genotypes for important agronomic parameters like grain yield, oil content, and oil production, all of which are influenced by genetic diversity. The current investigation determined genetic variation among studied sesame genotypes collected from KSA using molecular markers and cytological parameters to choose distinct and complementary parents for breeding and conservation strategy. The results have given helpful information about the genetic diversity of Saudi Arabia’s sesame cultivars. All the studied genetic diversity parameters recorded in this research showed that the ISSR markers utilized were highly reproducible. Moreover, the highest number of unique bands across the studied genotypes was exhibited by G6, while G4 scored the lowest. In the same context, cytological study agreed with molecular data and concluded that G6 was the most symmetrical (primitive) according to TF% and ASK %. While the genotype G1 was the most asymmetrical (advanced) according to A and A1 and had the highest number of unique bands from the ISSR marker.

## Data Availability

The original contributions presented in the study are included in the article/supplementary material, further inquiries can be directed to the corresponding author.
