# Supplementary material for: Genetic diversity using biochemical, physiological, karyological and molecular markers of Sesamum indicum L
Source: Front Genet. 2022 Oct 12;13:1035977. doi: 10.3389/fgene.2022.1035977 (PMC9597450; doi:10.3389/fgene.2022.1035977)
Supplement: Supplementary file 1 [file Table1.docx]

**Supplementary Table S1. Calculations and formulae of karyotype parameters.**

| **Karyotype parameters** | **formula** | **Reference** |
| --- | --- | --- |
| The total haploid length of the chromosome set (THL**)** | **(THL) = (LA+SA)** | [1] |
| Coefficient of Variation of Centromeric Index (CVCI**)** | **CVCI _=_** $\frac{\boldsymbol{SCI}}{\boldsymbol{XCI}}$ **x100** | [2] |
| Coefficient of Variation of Chromosome Length (CVCL**)** | **CV_CL_ =** $\frac{\boldsymbol{SCL}}{\boldsymbol{XCL}}$ **x100** | [3] |
| Mean Centromeric Asymmetry (MCA) | **M_CA_ = A × 100** | [4] |
| The Karyotype asymmetry index (AsK%**)** | **%AsK =** $\frac{\mathbf{LA}}{\mathbf{(LA+SA)}}$ **x 100** | [5] |
| The total form percent (TF%) | **TF % =** $\frac{\boldsymbol{\sum SA}}{\boldsymbol{\sum TL}}$ **x 100** | [6] |
| The of Karyotype symmetry (S%) | **S%=** $\frac{\mathbf{Mean length of the short arms}}{\mathbf{Mean length of the long arms}}\boldsymbol{x 100}$ | [7] |
| The intrachromosomal asymmetry index (A1) | **A_1_ = 1-** $\frac{\sum_{\boldsymbol{i=1}}^{\boldsymbol{n}} \frac{\boldsymbol{qi}}{\boldsymbol{pi}}}{\boldsymbol{n}}$ | [8] |
| The interchromosomal asymmetry index (A2) | **A_2_ =** $\frac{\mathbf{SCL}}{\mathbf{XCL}}$ | [9] |
| The degree of asymmetry of Karyotype (A) | **A =** $\frac{\sum_{\boldsymbol{i=1}}^{\boldsymbol{n}} \frac{\boldsymbol{pi-qi}}{\boldsymbol{pi+qi}}}{\boldsymbol{n}}$ | [10] |
| The asymmetry index (AI) | **AI =** $\frac{\boldsymbol{CVCL} \boldsymbol{X} \boldsymbol{CVCI}}{\boldsymbol{100}}$ | [11] |

**References**

1. Peruzzi L, I.J. Leitch and K.F. Caparelli (2009) Chromosome diversity and evolution in Liliaceae. Annals of Botany (London) 103: 459–475. doi: 10.1093/aob/mcn230
2. Paszko, A. (2006) A critical review and a new proposal of karyotype asymmetry indices. Plant Systematics and Evolution 258: 39–48. <https://doi.org/10.1007/s00606-005-0389-2>.
3. Peruzzi, L., Eroğlu H.E. (2013) Karyotype asymmetry: again, how to measure and what to measure? Comp Cytogen. 7(1):1–9. doi: 10.3897/CompCytogen.v7i1.4431
4. Arano H. (1963) Cytological studies in subfamily Carduoideae (Compositae) of Japan. IX. The karyotype analysis and phylogenic considerations on Pertya and Ainsliaea. Botanical Magazine (Tokyo) 76: 32–39. <https://doi.org/10.15281/jplantres1887.76.32>.
5. Huziwara, Y. (1962) Karyotype analysis in some genera of Compositae. VIII. Further studies on the chromosome of Aster. American Journal of Botany. 49, 116–119. <https://doi.org/10.1002/j.1537-2197.1962.tb14916.x>
6. Greilhuber, J., F. Speta (1976) C-banded karyotypes in the Scilla hohenackeri Group, S. persica and Puschkinia (Liliaceae). Plant Systematics and Evolution 126: 149–188. doi: <https://doi.org/10.1007/BF00981669>
7. Romero Zarco, C. (1986) A new method for estimating karyotype asymmetry. - Taxon 35: 526-530. <https://doi.org/10.2307/1221906>
8. Watanabe, K., T. Yahara, T. Denda and K. Kosuge (1999) Chromosomal evolution in the genus Brachyscome (Asteraceae, Astereae): Statistical tests regarding correlation between changes in karyotype and habit using phylogenetic information. Journal of Plant Research 112:145–161. https://doi.org/10.1007/PL00013869
